# Factors Predicting Patient Dissatisfaction 2 Years After Discectomy for Lumbar Disc Herniation in a Chinese Older Cohort: A Prospective Study of 843 Cases at a Single Institution

**DOI:** 10.1097/MD.0000000000001584

**Published:** 2015-10-09

**Authors:** Hui Wang, Di Zhang, Lei Ma, Yong Shen, Wenyuan Ding

**Affiliations:** From the Department of Spine Surgery, The Third Hospital of HeBei Medical University, Shijiazhuang, China.

## Abstract

We aim to identify factors predicting patient dissatisfaction 2 years after discectomy for lumbar disc herniation (LDH) in a Chinese older cohort.

Preoperative and 2-year follow-up data for 843 patients were analyzed. After 2 years of discectomy, the patients rated their satisfaction by Patient Satisfaction Index (PSI), with response of 1 or 2 defining satisfaction and a PSI response of 3 or 4 defining dissatisfaction. Associations between perioperative variables and satisfaction with the results of surgery were examined in univariate and multivariate analysis.

Six hundred fifty-seven patients had a PSI of 1 or 2 and were enrolled as satisfied group, 186 patients had a PSI of 3 or 4 and were enrolled as dissatisfied group. At baseline, no significant differences were found between the 2 groups in age, occupation, Oswestry Disability Index (ODI), Visual Analog Scale (VAS)-leg, and VAS-back. Compared to satisfied group, dissatisfied group had a significantly higher BMI and a higher incidence of depression. Two years after discectomy, no significant differences were found between the 2 groups in decrease of ODI, decrease of VAS-back, decrease of VAS-leg, surgery complications. Compared to satisfied group, dissatisfied group experienced higher incidence of symptom recurrence and depression. Logistic regression analysis showed that obesity, pre- and postoperative depression, symptom recurrence were independently associated with patient dissatisfaction 2 years after discectomy.

In conclusion, more than 70% patients expressed satisfaction with discectomy for LDH. Two factors could predict patient dissatisfaction and be assessed before surgery: obesity and preoperative depression. Symptom recurrence and postoperative depression are also associated with diminished patient satisfaction.

## INTRODUCTION

Lumbar disc herniation (LDH), a very prevalent spinal disease, is responsible for most low back pain. More than that, LDH can also lead to defecation dysfunction or even paralysis if accompanied by cauda equina injury.^1^ Discectomy is indicated primarily for treating LDH and has several advantages over intervertebral fusion techniques; these include simpler surgical procedure, shorter operation time, faster postoperative recovery, lower surgery cost, and preservation of mobility of the surgery segments.^2^

Many previous studies have shown satisfactory outcomes of the discectomy procedure for treatment of disc herniation, but there were still some dissatisfied cases.^3^ It is known that the satisfied and dissatisfied patients behave differently. Satisfied patients are more likely to cooperate with their healthcare providers by disclosing important medical information and continue using medical care services. On the contrary, the dissatisfied patients may make treatment less effective, either by neglecting to seek care when needed or refusing to comply with the prescribed course of treatment.^4^ Since the therapeutic effects are significantly influenced by patient satisfaction, it is reasonable to believe that spinal surgeons should strive to satisfy their patients as well as to provide the right and effective treatment.

To the best of our knowledge, little study focused on the association between perioperative factors and the patient satisfaction after spinal surgery, especially in older patients. Therefore, the primary objective of this study was to identify factors predicting patient dissatisfaction after discectomy for LDH in a Chinese older cohort.

## MATERIALS AND METHODS

### Subjects

We conducted a prospective study of 882 patients underwent discectomy from January 2008 to December 2011. The study was approved by Ethics Committee of The Third Hospital of HeBei Medical University. The primary inclusion criteria were the following: presence of mechanical back and radicular leg pain due to LDH; the radicular pain unresponsive to conservative treatment; magnetic resonance imaging (MRI) findings of LDH. The exclusion criteria were the following: spinal mechanical instability, an extra-spinal cause of back pain and leg pain; the presence of infection, trauma; unwillingness to participate in the study. All patients were provided written informed consent to participate in this study before the enrollment.

### Study Variables

The following data were collected at baseline, before the discectomy: age, gender, body mass index (BMI), occupation, felt depressed, Oswestry Disability Index (ODI), Visual Analog Scale (VAS)-leg, and VAS-back.

Two years after discectomy, the patients were contacted for a clinical evaluation, and the following were collected: satisfaction, surgical complications, symptom recurrence, felt depressed, decrease of ODI, decrease of VAS-leg, decrease of VAS-back. We chose a 2-year follow-up interval because we wished to study satisfaction at a time when outcomes were expected to be optimal.

### Methods Used to Evaluate the Study Variables

In each patient, the study variables were collected by a study personnel, not the operating surgeon, to avoid biased response. At baseline before the discectomy, the patient completed a questionnaire. The 2-year follow-up evaluation started with the administration by the personnel over the telephone, all the patients were asked to complete a telephone questionnaire to obtain an assessment of patient satisfaction and functioning.

A Patient Satisfaction Index (PSI)^5^ response of 1 or 2 was considered to indicate a satisfied outcome and a PSI response of 3 or 4 to indicate a dissatisfied outcome (Table 1). The Visual Analog Scale (VAS) consists of a horizontal line 100 mm in length, with the end points “No pain” and “Worst imaginable pain” placed at each end of the line. Participants were asked to make a mark on the line that best represents the level of pain intensity that they were experiencing 1 day before discectomy. The 2-year follow-up evaluation of back/leg pain was completed in the same way with the help of the study personnel. The Zung Self-Rating Depression Scale (ZSRDS)^6^ is a short self-administered survey to quantify the depressed status of a patient. There are 20 items on the scale that rate the affective, psychological, and somatic symptoms associated with depression, including 10 positively worded and 10 negatively worded questions. Each question is scored on a scale of 1 through 4 (based on these replies: “a little of the time,” “some of the time,” “good part of the time,” “most of the time”). Scores on the test range from 20 through 80. The scores fall into 4 ranges. A ZSRDS response of range I or II was considered to indicate nondepression and a ZSRDS response of III or IV to indicate depression (Table 2). Patients were divided into mental workers and manual workers according to their occupation. The patients, who undertook a small amount of manual labor and worked mainly indoor, were regarded to be mental workers, such as company employee, civil servant, teacher, bank employee, designer, government official, executive, etc. The patients, who undertook a large amount of manual labor and worked mainly outdoor, were regarded to be manual workers, such as construction worker, farmer, driver, etc.

**TABLE 1:**
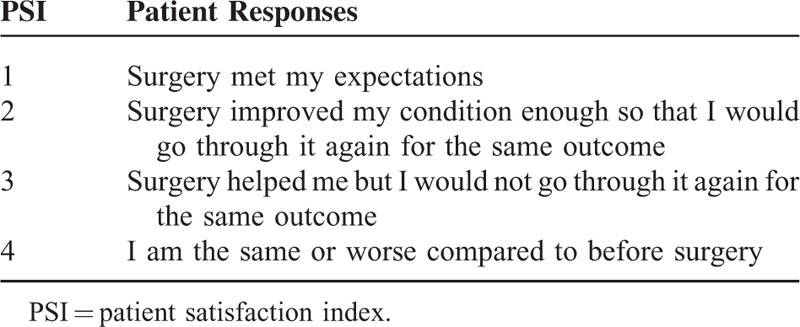
Patient Satisfaction Index (PSI)

**TABLE 2:**
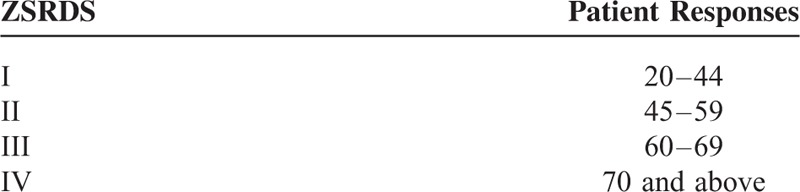
Zung Self-Rating Depression Scale (ZSRDS)

### Statistical Analysis

For the comparative analysis with patient dissatisfaction as the dependent variable, we performed univariate analyses using the Chi-square and unpaired *t* test. Variables with *P* values smaller than 0.05 in the univariate analyses, as well as a number of variables selected by experts, were entered into a multivariate logistic regression model. For each variable, we computed the odds ratio (OR) with its 95% CI. All statistical analyses were done using SPSS software version 13.0 (SPSS, Inc., Chicago, IL, USA).

## RESULTS

The cohort of patients evaluated on the day before discectomy comprised 882 patients. After 2 years, 25 patients were lost to follow-up, 8 refused the second evaluation, and 6 had died. Thus, there were 843 patients left and were divided into 2 groups with 657 patients (77.9%) in satisfied group showing 1 or 2 stage in PSI and 186 patients (22.1%) in dissatisfied group showing 3 or 4 stage in PSI.

At baseline, there were no significant differences between the 2 groups in age, gender, occupation, ODI, VAS-leg, and VAS-back. Compared to satisfied group, dissatisfied group had a significantly higher BMI and a higher incidence of depression before the operation (Table 3).

**TABLE 3:**
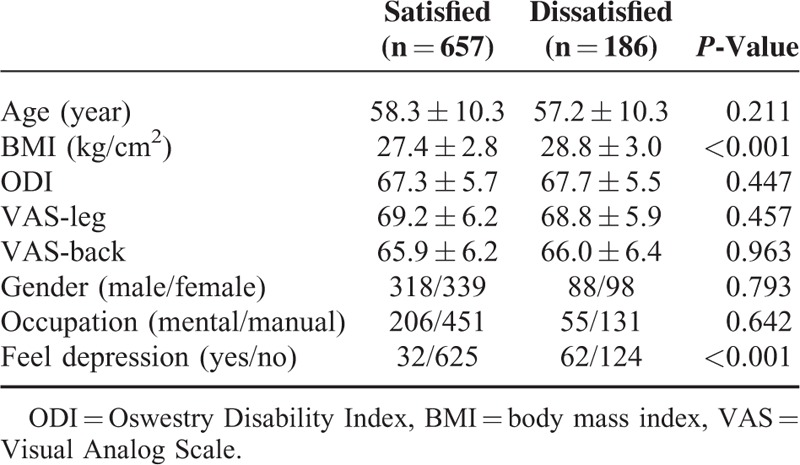
The Main Features in the Satisfied and Dissatisfied Patients at Baseline, Before the Surgery

Two years after discectomy, no significant differences were found between the 2 groups in decrease of ODI, decrease of VAS-leg, decrease of VAS-back, and surgery complications (Tables 4 and 5). Compared to satisfied patients, dissatisfied patients experienced higher incidence of symptom recurrence and depressive disorder 2 years after discectomy (Table 5).

**TABLE 4:**
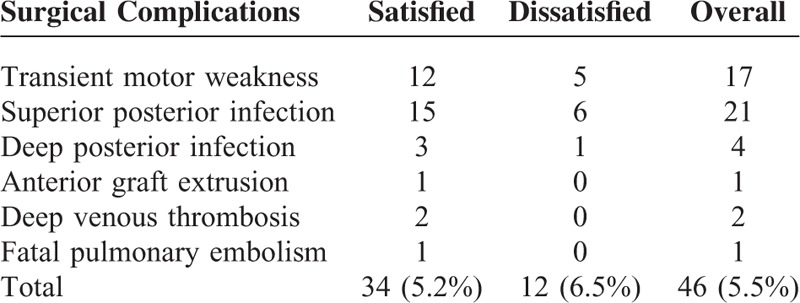
Surgical Complications in the Satisfied and Dissatisfied Patients After Discectomy

**TABLE 5:**
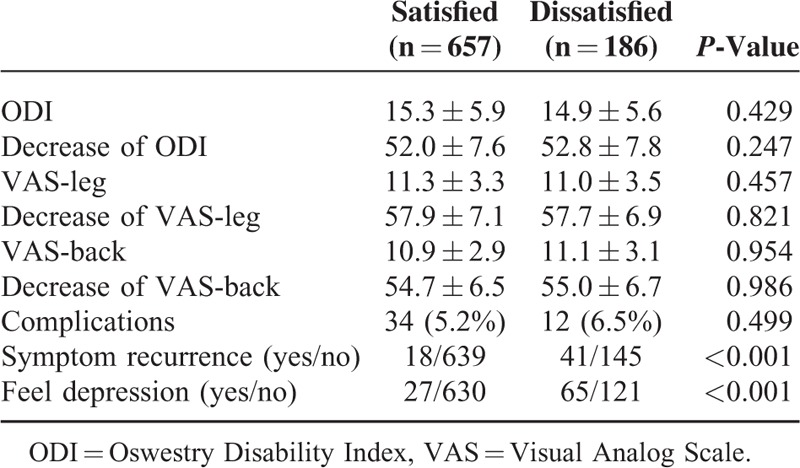
The Main Features in the Satisfied and Dissatisfied Patients at 2-Year Follow-Up Evaluation

The following variables were entered into the multivariate model: BMI, preoperative depression, VAS-leg at baseline, ODI at baseline, VAS-back at baseline, surgery complications, decrease of ODI, decrease of VAS-leg, decrease of VAS-back 2 years after discectomy, symptom recurrence, postoperative depression. When included in a multivariate logistic regression model, obesity, preoperative depression, postoperative depression, symptom recurrence were independently associated with patient dissatisfaction 2 years after discectomy (Table 6).

**TABLE 6:**
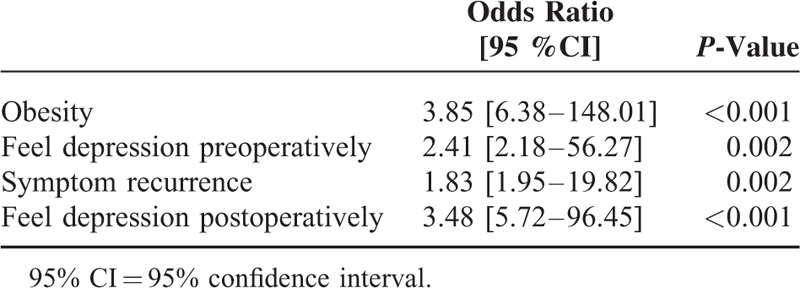
Factors Predicting Dissatisfaction After Discectomy, Identified by Multivariate Analysis

## DISCUSSION

In the present study, the majority of patients were satisfied with the results of discectomy and would have the procedure again knowing what their outcome would be. However, 22.1% of the patients were not satisfied according to the PSI evaluation, suggesting that surgical indication remains to be improved. We found that both obesity and depression were significantly and independently associated with diminished patient satisfaction, and can be assessed before surgery. Symptom recurrence and postoperative depression were also associated with diminished satisfaction. These results were not confounded by other variables potentially affect satisfaction such as age and gender.

Many previous studies performed by different medical center have proved the adverse effects of depression on postoperative patient satisfactory degree. Linn et al^7^ reported that the psychological status strongly influenced the patient reported satisfaction ratings in internal medicine practices. Bui et al^8^ reported that depressive symptoms were associated with patient dissatisfaction 12 months after a breast cancer diagnosis. Similar to the previous reports, our study revealed that both preoperative and postoperative depression predicted poor satisfaction 2 years after discectomy. In China, most of patients from rural areas are likely to be doubtful about the operation curative effect, and the patients often present with long duration of symptoms coupled with anxiety before surgery, this may increase the preoperative depressed emotion. More importantly, for many rural families, the surgery cost can be the equivalent of 1 or 2 year's income, most of the patients have great expectations for the operation, and they hope to live like normal people after discectomy. Sometimes it may be unrealistic, because not all the patients can access significant improvement of neurological function or pain relief. If postoperative recovery does not meet the patients’ expectation, the preoperative depressed emotion may continue or aggravate. What's worse, some patients without depression before operation may even suffer from depression after the treatment and the subjective satisfaction may decline due to the depression. These patients represent a unique group in which psychological factors, especially the feeling of depression, may play a particularly important role in their perception of overall satisfaction, despite surgical effectiveness, and functional improvement.^9^ Therefore, for patients that felt depression preoperatively and without neurological compromise requiring prompt surgical intervention, initiating conservative therapy may be the best option to improve patient overall satisfaction. While for the patients with depression firstly found after the operation, psychotherapy may be helpful.

Relationships between patient satisfaction and obesity have been confirmed in total knee replacement, total knee arthroplasty for osteoarthritis,^10–12^ and surgery for lumbar spinal stenosis.^13^ For patients undergoing discectomy for treatment of LDH, our result also confirms the widely held impression that obesity is a risk factor for poor postoperative satisfaction. There are 2 hypotheses. First, it is assumed that many healthcare providers have a negative attitude toward obese people, and they often view obese patients as lazy and lacking motivation.^14,15^ Due to the general stigma of obesity, obese patients may enter the hospital expecting to have to face discrimination based on their weight,^16^ which may increase ZSRDS. Second, there is substantial evidence that obesity is associated with reduced functioning and decreased range of motion. Although no statistical difference was found in ODI at baseline and decrease of ODI 2 years after discectomy between satisfied and dissatisfied group, the direct influence of obesity on general activities, such as difficulty with postoperative mobility may reduce the effectiveness of surgery. However, obese patients should not be excluded from the benefit of surgery, given that their overall improvements of ODI were equivalent to those of patients with a lower BMI.

Another adverse factor on patient dissatisfaction is symptom recurrence, which brought the physical and mental impairments to the patients so that the patients are very painful, and difficult to accept. Sebastian et al^17^ noted that recurrence after operations of disc herniation using only discectomy cannot be prevented, the rate of recurrence is reported variously ranges from 5% to over 20%. Symptom recurrence after an initial period of symptomatic relief can be caused by a true recurrence of disc herniation at the same disc level, new disc herniation at a different disc level (adjacent disc herniation), epidural fibrosis, arachnoiditis, foraminal stenosis, or segmental instability.^18^ X-ray, CT, and MRI examination may be helpful for the doctor to make up the therapeutic program. Sometimes revision surgery is inevitable. Patients requiring revision spinal surgery often present with severe symptoms coupled with anxiety regarding previous discectomy, which they perceived to be treatment failures. Although recent experience indicates that the outcome of revision surgery for treatment of relapsed disc herniation is favorable, many of the patients do not have the courage to accept the second operation and noted that they would not go through the first surgery again for the same outcome if possible. This may lead to increase in the incidence of postoperative depression and decrease in satisfaction degree. However, 18 of the 649 patients in satisfied group experienced symptom recurrence, but expressed satisfaction to the discectomy. There are 2 possible reasons. First, one thing that they had in common was the good education; they got a full understanding of the natural course of LDH and the prognosis of discectomy. Second, another thing that they had in common was optimistic personality; they were more likely to cooperate with us by disclosing their discomfort and continue using medical care services.

There are some limitations to this study. First, satisfaction is believed to be an attitudinal response to value judgments that patients make about their clinical experience and is associated with many variables, such as patient symptom characteristics, symptom-related expectations, functional status, mental disorders, unmet expectations, doctor–patient communication, and so on. In this study, only a small portion of variances were chose in predicting satisfaction and selection bias may exist. Second, the satisfaction is evaluated by PSI and the patient psychological situation is evaluated by ZSRDS, which are easy to use, but are quite simple. However, we report the first prospective study to evaluate outcomes of discectomy in a large Chinese cohort of patients with LDH. We identify 2 factors that predict patient dissatisfaction and can be assessed before surgery: obesity and felt depression, while postoperative feeling depression and symptom recurrence are another factors associated with patient dissatisfaction 2 years after discectomy. Our results indicate that these factors should be addressed regarding the selection of the operative treatment method and may assist spinal surgeons in customizing patient-specific estimates of the likelihood of successful surgery.
